# Application of the Metabolomics Approach in Food Authentication

**DOI:** 10.3390/molecules26247565

**Published:** 2021-12-14

**Authors:** Jinap Selamat, Nur Amalyn Alyaa Rozani, Suganya Murugesu

**Affiliations:** 1 Faculty of Food Science and Technology, Universiti Putra Malaysia, Serdang 43400, Malaysia; amalynalyaa@gmail.com; 2 Institute of Tropical Agriculture and Food Security (ITAFoS), Universiti Putra Malaysia, Serdang 43400, Malaysia; suganya@upm.edu.my

**Keywords:** food authenticity, metabolomics approach, mass spectrometry, chromatography, spectroscopy

## Abstract

The authentication of food products is essential for food quality and safety. Authenticity assessments are important to ensure that the ingredients or contents of food products are legitimate and safe to consume. The metabolomics approach is an essential technique that can be utilized for authentication purposes. This study aimed to summarize food authentication through the metabolomics approach, to study the existing analytical methods, instruments, and statistical methods applied in food authentication, and to review some selected food commodities authenticated using metabolomics-based methods. Various databases, including Google Scholar, PubMed, Scopus, etc., were used to obtain previous research works relevant to the objectives. The review highlights the role of the metabolomics approach in food authenticity. The approach is technically implemented to ensure consumer protection through the strict inspection and enforcement of food labeling. Studies have shown that the study of metabolomics can ultimately detect adulterant(s) or ingredients that are added deliberately, thus compromising the authenticity or quality of food products. Overall, this review will provide information on the usefulness of metabolomics and the techniques associated with it in successful food authentication processes, which is currently a gap in research that can be further explored and improved.

## 1. Introduction

Food authentication is an analytical process that is used to verify and ensure that food production follows the label description based on the regulations. Food authenticity, as defined by the International Food Authenticity Assurance Organization (FAAO), is a technical method to validate the declared and presented product information on genuine food ingredients and sources that are verified by authority. The process has significant impacts on food quality and safety due to the wide global supply chain and its contribution to food fraud. Food authenticity is also necessary to ensure compliance with national law, international standards, and other directives [1]. For this reason, authenticity testing is required to prove that food products’ content is legitimate and safe for consumption. 

**Figure 1:**
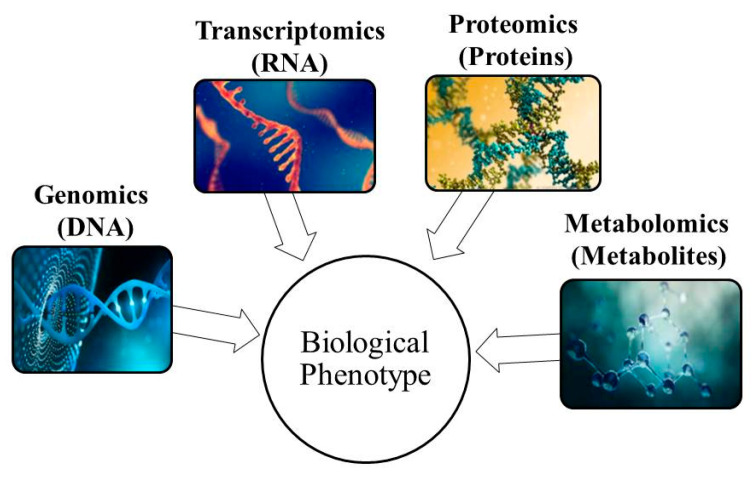
Omics approaches applied in the identification of the biological phenotype of various biological samples, including food commodities.

Metabolomics is one of the four “omics” technologies (Figure 1). It is a technical and systematic approach that involves the study of metabolite concentrations and interactions in organic entities. The approach simultaneously aims to detect the overall changes in metabolites in bio-systems involving microorganisms, plants, animals, and humans, which directly reflects the underlying biochemical activities occurring in the cells, tissues, organs, and organisms. Metabolomics is used in various fields, including pharmaceutical, food, agriculture, etc. Food metabolomics involves the application of metabolomics in food resources, production, and processing, as well as in human diets. The application of food metabolomics has gradually increased and made significant breakthroughs in recent years, along with the revolution in the food industry [2,3,4].

Current research studies applying metabolomics in the food industry mainly involve the use of various robust technologies and routine analytical techniques have been used to determine food identity markers. These instruments have a large impact on the search for metabolic markers of food authenticity, nutritional quality, and the identification of adulteration in food [5,6]. The application of metabolomics in food commodities for authentication purposes has had significant effects [7]. Technically, the metabolite profile of the nutritional content of tested food products can be achieved using a metabolomics study, which covers a wide range of targeted and untargeted metabolomes using analytical technologies and incorporates chromatography and spectroscopy instruments with statistical analysis (multivariate data analysis). The interpretation of the raw data of known and unknown metabolomes is generally conducted based on several databases, such as the Food Component Database (www.foodb.ca, accessed on 5 December 2020), the Human Metabolome Database (www.hmbd.ca, accessed on 24 November 2020), and PhytoHub (www.phytohub.eu, accessed on 18 November 2020). 

In recent times, the application of metabolomics in food has been shown to potentially have a greater impact and contribute to agriculture, food industries, and human health. This review provides comprehensive information on the application of the metabolomics approach to food authentication. The review details the recent techniques and instruments utilized in the food industry for authentication purposes and the food products analyzed through the metabolomics approach. This review will benefit food personnel and researchers working on food authentication and adulteration detection, which defines food quality and safety.

## 2. Food Authentication

Food authentication is regarded as a crucial part of the food industry that reflects food quality and safety. With an increasing concern among consumers globally, the authentication field is rapidly growing and expanding through many reliable and efficient technologies that are used to comply with the demands and standards set by authorities [1]. The authentication of food may affect many deceptive processes, such as the mislabeling of its origin, techniques of production, or forms of technical processing such as irradiation, freezing, and microwave heating. It is especially focused on revealing the unique characteristics of high-value goods; these products are often targets for deceptive labeling because of their economic value. In recent studies, many efficient technologies, and instruments, including liquid chromatography (LC), Fourier transform infrared spectroscopy (FTIR), mass spectrometry (MS), and nuclear magnetic resonance (NMR), have been used to identify and detect emerging fraudulent trends in various food samples [1,8,9]. Food researchers have also engaged in the research and development of accurate and effective analytical techniques to identify food adulteration and fraud.

Various food products were analyzed and investigated for their claimed authenticity (Table 1). Some of the issues reported include, for the example, the inaccurate declaration of fruit type and the undeclared addition of water, sugar, acid, pulp wash, and peel extracts to fruit juice. The authenticity of grain, involving basmati rice being replaced with non-basmati rice, the discrimination of viable germinating corn and soybean, and issues with the geographical and cultivator origin of the grain, were highlighted in a previous study. The authentication of milk and dairy products includes yogurt and cheese production, as well as the determination of milk itself. In oils and fats, authenticity testing often reveals the undeclared addition of oils to other oil and butter adulterations. As for livestock products, issues including incorrect declaration, mislabeling, undeclared water addition, and discrimination between fresh and thawed meat were identified during the process [10]. This indicates the need for appropriate and reliable analytical methods to ensure how food producers meet industry needs and protect the public from misleading or fraudulent labeling and avoid food safety issues.

## 3. Metabolomics Approach 

The goal of metabolomics is to investigate multiple metabolites in a cell, tissue, or organism. The untargeted nature of metabolic applications in food authentication allows the detection of emerging fraud [8]. Metabolomics offers a snapshot of relevant biological processes by evaluating global amounts of small molecular metabolites (amino acids, organic acids, starch, fatty acids, lipids, hormones, peptides, and vitamins). It provides a read-on of the metabolic activity status for genetic variation, genetic expression, or external factors, including infection and allergens, when a particular metabolomics profile recognizes the interactions between the host molecules and environmental agents, namely deoxyribonucleic acid (DNA), ribonucleic acid (RNA), proteins, lipids, and other enzymes. On the other hand, metabolomics offers a snapshot of the host’s physiology and its environmental reaction, which can also be related to the resulting phenotype (safe vs. disease) and endotype [14]. Therefore, metabolomics combines high-throughput analytical techniques with bioinformatics methods to provide information about a wide range of metabolites, as well as the potential for host susceptibility biomarkers, environmental risk factor response assessments, monitoring of the possible development of chronic asthma, and wheeze. It also offers the potential to illustrate biological pathways [15].

### 3.1. Principle

Metabolomics has provided a systematic study of small molecules in a biological system, offering mechanical information on physiological and pathological processes in distinct scenarios [16]. A systematic approach to metabolomics has achieved tremendous success in resolving a range of problems, not only in food fraud or authentication, but also in biological, biomedical, agricultural, and nutritional science, including drug discoveries, disease diagnoses, and plant phenomena [17]. 

This approach is generally classified into two: untargeted and targeted metabolomics. Non-targeted metabolomics is said to be an extensive analysis that consists of chemical unknowns of all detectable analytes in one sample. There is limited knowledge of which metabolites are detected before the gathering of data. Due to their extensive nature, untargeted metabolomics must be combined with advanced techniques of chemometric analysis. This can reduce the comprehensive data sets into a smaller collection of controllable variables. Despite recent technological advancements, the main disadvantages of unspecified metabolites are the time required to process large amounts of raw data, difficulties in identification, classification, and intrinsic analytic platform coverage [15]. This untargeted approach therefore poses extreme challenges to the identification and detection of metabolites, and further research is required to examine the effect of this approach on the climate [15,18]. 

For targeted metabolomics, the chemical attributes of the metabolites to be tested are identified before data acquisition occurs, and analytical methods are designed to provide high precision, selectivity, and reliability. This method is established using true chemical principles [19]. Once the chemical identity of metabolites is known, the subsequent process of deriving biological information from the acquired data can be started immediately after a complete overview. The targeted approach makes use of knowledge of a wide range of metabolic enzymes, their kinetics, end products, and biochemical pathways that have been identified [20]. So far, metabolomics tests have been conducted either with MS or NMR [18]. NMR is capable of measuring metabolite levels in intact tissue, while instruments equipped with an MS analyzer provide a wider detection scope comprising metabolites of either a polar or a non-polar nature [21,22]. Consecutive targeted and untargeted approaches should be used to reliably classify and measure metabolites accurately.

Untargeted metabolomics is more comprehensive, unbiased in detecting high abundance molecule, offers a high throughput, and enables the discovery of new compounds. It is a form of hypothesis generation that is focused on global or comprehensive detection, and the relative quantification of small molecules in a sample [23]. However, it is possible to obtain high rates of false positives and false negatives, which lead to difficult data interpretation. Meanwhile, targeted metabolomics offer simpler data interpretation, and the data can be linked to a pathway. However, it can target only a limited number of compounds, and untargeted compounds cannot be assessed. It is hypothesis-driven, which enables validation, and focuses on the absolute quantification of well-defined metabolites [23,24,25,26,27]. The advantages and disadvantages of the metabolomics methods are tabulated below (Table 2).

### 3.2. Challenges of Metabolomics Approach

Metabolomics is one of the newest innovations that have been rapidly introduced in many scientific fields. Most recently, metabolomics studies have provided a broad range of new metabolites and more specific biological characteristics for many species. Despite these dramatic advances, we are far from obtaining thorough coverage of all metabolites [28]. In addition to the applications of metabolomics in medical science for the discovery of a variety of biomarkers for disease risks, including for diabetes, heart disease, and cancer [27,28,29], metabolics studies have also revealed the metabolomics pathway disruption in many food authentications, including meat [30,31], fruit [26,32], milk [33,34], and honey [35]. The verification of food authenticity raises public confidence in food ingredients and manufactured food components. The metabolomics approach not only allows metabolic identification via analytical methods such as chromatography (liquid or gas) coupled with MS but also directly evaluates the nutritional composition of food. Carbohydrates, proteins, and fats, as well as their primary components, can be determined if combined with hydrolysis procedures [7]. This knowledge is important for customers and food producers, who are interested in possible health benefits and nutritional value. Metabolomic approaches are therefore expected to become a powerful instrument of authentication of food and food adulteration discovery.

Although the analysis of the metabolics and mechanisms of diseases may be proven to be effective by the metabolomics approach, some obstacles need to be considered when conducting a metabolomic analysis, such as the sensitivity of metabolites in the existing environment, the choice of the instrument used, and the cost of the analytical instrument. Furthermore, the metabolome is biologically responsive to multiple genetic and environmental stimuli. In terms of their stability, metabolites are indeed different and have very different turnover rates within cells. Others may be volatile in the presence of oxygen, light, or various temperatures, or under other analytical conditions in which the tests are conducted that may cause significant problems. The preparation of samples for the detection of vitamins, for example, is quite sensitive due to their photosensitive nature, which may trigger degradation upon direct contact with light [15]. Therefore, there is no universal analytical method available at present because of this wide-ranging diversity, which has the sensitivity and specificity to identify and quantify all existing metabolites in the wide range of commonly used biological samples [29]. Metabolites also require different instruments to identify the required compound in complex mixtures based on their nature and stability. The choice of the instrument may also be challenging in the identification of metabolite compounds. The choice of which platform to use can be troublesome for a number of different types of instruments, since no one platform can disclose the entire metabolome. This is because the chemical constitutions of the metabolites are heterogeneous, and each instrument features unique analytical restrictions. 

### 3.3. Statistical Analysis in the Metabolomics Approach

Generally, the metabolomics approach generates large data sets that require sophisticated analytical tools to analyze. Typically, univariate and multivariate statistics are applied in the analyses of metabolomics data, depending on the type of experiments performed. The univariate analysis provides a preliminary overview of the data’s characteristic potential for discrimination. Some of the statistical tests conducted include the t-test and volcano plots, as well as one-way analysis of variance (ANOVA) associated with correlation analysis depending on the tests and data requirement. In order to reduce false-positive results in metabolite identification, the Benjamini–Hochberg correction is usually carried out and determined using significant P-values. Meanwhile, multivariate data analysis (MVDA) is considered the ideal tool to analyze larger data sets of omics. Some of the techniques involved include regression analysis, principal component analysis (PCA), partial least square discriminant analysis (PLS-DA), and orthogonal partial least square discriminant analysis (OPLS-DA), the extension of PLS-DA. These analytical techniques cluster and discriminate the variables of the data observed and enable the detection of metabolites, be they targeted or untargeted [36]. 

MVDA applies the two methods, namely the unsupervised and supervised data analysis. The unsupervised exploration does not require any preliminary information on the sample characteristics to perform the modeling. Principal Component Analysis (PCA) and Hierarchical Cluster Analysis (HCA) are the two most commonly used unsupervised methods PCA extracts the dominant patterns from the data matrix consisting of variables called the principal components, which represent the variables in linear combinations. The latter are used to determine the differences or similarities between the samples observed. Discriminant analysis (PLS-DA and OPLS-DA) is a supervised method used for samples or variables discrimination and describes the classification information in one component [37].

## 4. Detection Technologies

The complexity of metabolites can be measured and detected using a broad variety of analytical methods. New approaches to metabolite analysis and detection are being built to help address some of the challenges involved with metabolomics analysis. The detection methods or instruments used in food authentication include high-performance liquid chromatography (HPLC), FTIR, chromatography coupled with MS, and NMR. While metabolomics aims to generate a database of all the metabolites found in tissue, no single analytical technique is capable of isolating and detecting all of the different molecules at the same time.

### 4.1. High-Performance Liquid Chromatography (HPLC)

The HPLC is commonly used for food authentication and is one of the most effective technologies at solving problems of food safety and ensuring that food is genuine to eliminate fraud. It is an analytical chemistry technique for the separation, detection, and quantification of ingredients in a mixture, which greatly depends on the solutes’ solubility and polarity in the solvent system used. Compound detection is usually performed using different types of detectors that can be used to detect chemical adulterants, including tocopherols, fatty acids, and oligosaccharides. The analytical methods using HPLC typically detect carbohydrates, amino acids, carotenoids, phenolics, and other organic compounds [38,39]. Various food products were analyzed using this system well before the advancement of technology. Fruit products are commonly analyzed using HPLC to identify and monitor the authenticity of phenolic compounds, organic acids, carotenes, amino acids, anthocyanins, and sugar. The greatest strengths of HPLC are its versatility and its applicability to different forms of analytes, from small organic molecules and ions to massive biomolecules and polymers. HPLC performs efficiently in practice due to the gentle, predictable design of liquid phase chromatographic processes and the availability of accurate instrumentation with effective and highly sensitive metabolite detection. Some of the advantages of using HPLC include the efficiency and reliability of this quantitative instrument and its superior detection accuracy (Table 3). It also provides the recovery of quantifiable samples and convenient analysis of different types of samples [37].

### 4.2. Fourier Transform Infrared (FTIR) Spectroscopy

FTIR spectroscopy is one of the most widely used screening methods for food fraud and authentication in both industry and government laboratories. With minimal sample preparation is required, the analysis is considerably quick and non-destructive to the sample; thus, FTIR has emerged as an appealing alternative to conventional analytical methods for analyzing samples that are both cost- and time-effective. It has received much attention for use in quantitative studies of edible fats and oils [39]. Previous research has shown that FTIR spectroscopy can be used to analyze pork in beef ball formulations and juice concentration products. The instrument was also used to detect adulteration in virgin oil [40,41,42]. 

Recently, Rohman [43] approached the use of FTIR spectroscopy coupled with chemometric techniques to detect food authentication in meat and meat products. Moreover, this method has also been applied to other food products, such as herbal food, agricultural products, and dairy products [39]. Valand et al. [44] pointed out that FTIR is typically used as an analytical technique for the detection of organic, polymeric, and inorganic materials in some situations. Infrared light is used to screen test samples and to observe chemical properties in the FTIR analysis process. The resulting signal on the spectrum of the detector is a molecular fingerprint of the sample, usually ranging from 4000 cm^−1^ to 400 cm^−1^. A unique spectral fingerprint is created by each molecule or chemical structure, which makes FTIR analysis a great chemical identification tool. In general, the amount of material needed for viable analysis is very small and with low sample preparation, most analyses can be performed reasonably quickly. Furthermore, this method is also effective and easy to carry out without the need for sample pre-treatment. These methods provide quick and reproducible means of handling food products, be they in solid, liquid, or paste form, with non-destructive tests, which typically take less than five minutes for sampling or analysis [45].

Rohman [43] described the benefits and limitations of FTIR spectroscopy (Table 3). FTIR is shown to have a high sensitivity and high speed in the detection of various peaks corresponding to various metabolites. It also provides all frequencies to measure metabolites simultaneously and is efficient in data interpretation. Even so, it is difficult to analyze aqueous solution with this instrument which means that it cannot identify molecules that contain two identical and symmetrical atoms, such as nitrogen and oxygen.

### 4.3. Nuclear Magnetic Resonance (NMR) Spectroscopy

NMR is yet another analytical technique used to identify metabolites in various food products, such as fermented food, honey, milk, meat, and coffee products. Some resonance peaks are marked as spectral intensities and frequencies (chemical changes), typically shown in portions per million (ppm) of the NMR carrier frequency, for instance, 400 MHz [46]. Ultimately, the concentration of compounds in complex mixtures can be defined and evaluated via these peaks without the need for extensive sample preparation. NMR is thought to offer the primary benefit of not requiring complicated sample preparation, and in a single experiment, it is possible to determine very different chemical species. NMR spectroscopy is relatively easy, nearly every combination of compounds can be resolved when using it, and most important of all, almost every food component can be detected with a quantitative signal. Furthermore, NMR is a very responsive and high-performance technology. However, analysis of compounds is time-consuming, costly, and sometimes challenging in these conditions when attempting to perform risk assessment. Much research is now concentrated on developing effective instruments that can assess the existence of non-target risks quickly, and NMR is arguably the leading platform to solve this, despite its minimal sensitivity [8,21,22,46]. However, the application of ^2^H (deuteron), ^13^C, and ^15^N serve as ideal metabolic tracers in detecting nuclei present at low abundance [47]. Recently, various strategies have been established to improve NMR sensitivity. These include the introduction of higher field magnets that operate at a frequency higher than 1.2 GHz, improvised NMR probes that are cryogenically cooled, hyperpolarization, micro coil probes, and superconducting coils. These potential strategies increase the sensitivity of detection mainly for low abundance analytes while being cost-effective [48].

### 4.4. GC-MS and LC-MS

Mass spectrometry (MS) for the study of metabolites in plants and animal tissues is used in conjunction with chromatographic techniques involving both gas and liquid mediums. This technique produces unique chemical fingerprinting that separates or verifies food. Cao et al. [49] explained that only after converting the molecule into a gas-stage ion can a mass spectrometer determine the mass of a molecule. To achieve this, the electrical charge is transferred to the molecules and the electrically charged ion flow is converted to the proportional electrical current read by the data device. The data system translates the charge and exposes it to digital material as a mass spectrum. The unknown compounds can be identified while the known compounds can be quantified, and the determination of the structure and chemical properties of molecules can be performed using the classification of the molecular weight by the mass spectrometer.

The components of a mixture are separated by GC or LC, and the MS detects and characterizes each component. The MS-based methods feature performance characteristics that are suitable for sample types in high-performance applications of food metabolomics [50]. However, MS features the drawbacks of its sampling material and its requirement of direct contact with its broader instrumentation. MS spectral resolution is more detailed; therefore, the fingerprints of food chemicals are more easily recognized. It also provides greater flexibility due to the exchangeability of its ion source. MS can create numerous ionizations with different ion sources and can calculate chemical compounds with different ion sources [51]. In recent years, MS has concentrated primarily on instrumental changes to achieve higher mass resolution, accurate mass measurement, higher sensitivity, improved fragmentation, and more linearity.

The method of GC-MS has been implemented in metabolomics for a considerable time due to its high spread capacity and robustness and is particularly suitable for testing volatile organic compounds and primary derivatives [52]. Generally, GC is used frequently in food adulterant identification and the quantitative and qualitative evaluation of products, food additives, food adulterants, and hazards, to detect nutritional quality and to improve food protection and implement various food varieties. For instance, oil adulteration and blending composition can be determined using GC-MS through the detection of triacylglycerols, sterols, and fatty acids. Technically, fatty acid (methyl ester) characterization and differentiation can be achieved using GC-MS [37]. GC also has been used to identify metabolites and to study the qualities of potato tuber in barley seeds, ground beef, and chicken [52]. Detection using GC-MS is made easier by in-house validated databases (Wiley and NIST 14 library) of volatile compounds that can be used to identify metabolites using the mass spectral method [53]. 

Furthermore, LC-MS is ideally suited to analyze polar compounds. The distinction between GC-MS and LC-MS is that GC-MS uses inert gases like helium, while LC-MS uses solvents of the same capacity as its mobile phase. LC-MS is a combined analytical chemistry technique, which is applied to distinguish multiple components mixtures and to provide the molecular identity of the individual components with high molecular sensitivity and specificity. Nevertheless, the quantification of certain chemical substances can be difficult for foodstuffs with extremely complex matrices, especially because of their potential to interact with an unknown agent [54]. 

The method of LC equipped with high-resolution mass spectrometry provides a wider scope of analytes and fragmentation details for accurate identification. The types of ion source and mass analyzer used may improve the spectral data obtained from the analysis. Electrospray ionization source (ESI) is a robust ion source that can detect a wide range of metabolites, including peptides. Furthermore, Q-ToF, which stands for quadrupole time-of-flight analyzer, improves the limits of detection of targeted metabolites with high mass accuracy, thus allowing the determination of molecular formulas for small molecules [55]. This eases the identification of some low-abundance molecules in the food matrix that are often neglected during quality control analyses. LC-MS is widely used to determine the geographical origin and distinguish the cultivation methods of certain agricultural products, such as maize and grains, based on their metabolome [56]. Recently, more attention has been paid to detecting and quantifying food allergen proteins [57]. 

Apart from the GC- and LC-MS platform, other techniques that are used to assess food quality employ MS. Direct analysis in real-time (DART) is an ionization technique that is rapid and robust with minimal sample pre-treatment. It fundamentally relies on atmospheric pressure chemical ionization. DART coupled with MS is sought as a high-throughput tool for both the targeted and non-targeted analysis of food components in metabolomics fingerprinting or profiling. The instrument is also applicable in the quantification of low-molecular-weight metabolites, including trace materials or contaminants in food matrices [58]. High-resolution mass spectrometry (HRMS) is another application used in food safety to detect natural toxins, including mycotoxins, pesticides, and veterinary drug residues. Studies have reported the detection of new fungal metabolites, contaminants, and various food adulterants [59]. 

Compact portable membrane inlet MS (MIMS) is another monitoring system that is employed to detect and monitor mycotoxins in food matrices. The first investigation using this system was carried out to detect volatile emissions of grape berries infected by the fungus *Aspergillus carbonarius*, which results in different olfactory chemical profiles that can be used on-site to detect and monitor toxicity levels, which may consequently improve the food safety aspect [60]. Another portable MS-coupled probe is the direct inlet membrane (DIM) probe, which can be used to directly analyze the traces of active fragrant or essential oil components present in complex mixtures in real-time. These portable probes are considered a novel in-situ analysis technique [61]. Apart from these portable MS detectors, a benchtop miniature of the MS system, named Mini 12, was developed. As an ambient ionization source with an MS analyzer, it is capable of producing quantitative data for small volume samples; it is thus able to trace chemical markers in food matrices [62].

Low-temperature plasma (LTP) provides ambient ionization with the desorption of molecules in all states of matter. The coupling of low-temperature plasma (LTP) probes with chromatographs and mass analyzers may directly detect metabolites in complex matrices without prior sample preparation. LTP is considered an efficient method with which to test food matrices for authentication purposes [63]. Analyses of vegetable oils demonstrated quality measures through the characterization of free fatty acids and volatile complex [64]. This probe is also applicable in the direct analysis of sensorial components of a volatile nature in food products such as coffee [65]. Due to its high selectivity and sensitivity, LTP-MS is mainly used in the detection and quantification of the illegal food additive, melamine, in complex foods such as milk powder, whole milk, fish paste, and urine [63,65,66]. 

## 5. Food Authentication Using Metabolomics Approach

In past decades, the metabolomics approach was widely used in the study of authenticity in different food commodities. Recent studies have been conducted to detect and evaluate the data on food authentication and adulteration. Some of the studies that have successfully applied metabolic technologies in food authentication are discussed in detail as per below.

### 5.1. Meat and Fish/Seafood Products

The primary focus of metabolic processes in the supply chain for characterizing meat and biomarkers is animal and meat muscle. Certain distinct variables related to genetic and sensory attributes are assessed to monitor meat quality. This can be achieved through the characterization of the potential biomarkers corresponding to the meat quality, using metabolomics analysis on the muscles and meat samples. Other factors that should be taken into consideration include the feed system and meat handling, which are generally associated with post-mortem storage, processing, and sanitation. Different approaches are used for quality control of meat, including MS, NMR, and FTIR. A study detected pork adulteration in beef balls by analyzing the fats obtained from the meatballs using FTIR. The results obtained were coupled with the chemometrics analysis applying the partial least squares (PLS) to measure the adulterant pork in the selected fingerprint area (1200–1000 cm^−1^),which allowed the authors to track the adulteration of beef meatballs with pork [41]. This method can therefore be used to establish a distinction between these fats. In order to investigate the lard in the meatball broth, FTIR with PLS and principal component analysis (PCA) was used. The lipids containing pork fat that were extracted were observed at wavenumbers of 1018–1284 cm^–1^. The outcome demonstrates that the presence of lard in meatball broth can be classified and quantified without any misclassification [67]. Further studies on the identification of horsemeat and pork in processed meat products have been performed using an HPLC-MS/MS-based method. The established peptide markers from various types of meat and meat products are thermally stable and can thus be detected in processed food with high precision and sensitivity. The evaluation of different extraction methods indicates that the modification and optimization of protein extraction protocol may affect the efficiency of the process [31]. 

Another study utilized LC-MS-based metabolomics analysis to obtain the metabolic profiles of upon-arrival slaughtered and dead chicken organs (muscle, heart, and liver tissue). The study distinguished both the chicken samples based on the biomarker profiles and discriminant analysis (MVDA). Based on the existing database, and chemical specifications from the LC-MS profile, a distinctive metabolome, namely sphingosine, was identified from the muscle tissue samples that may potentially identify fraudulently processed chicken meat. However, the markers cannot be detected in heart and liver extracts, so further work is necessary to verify this marker by using MS [68].

The establishment of a method for the identification of species in fish or seafood products is also a significant issue in food authenticity. Fish products are mainly composed of water, protein, fat, and other compounds present at low concentrations (mg 100 per g), such as vitamins and minerals, and the spectrum of the absorption obtained by the analytical instrument can be formed by all these components. Low-field-NMR (LF-NMR), which works within the 2–25 MHz frequency range and provides a simpler and cheaper NMR traditional spectrometer version, is used in fish products [69]. Research using LF-NMR spectroscopy was carried out by Gudjónsdóttir et al. [70] to determine the effects of various pre-salting methods on denaturing proteins and changes in the muscle properties of dry, salted cod cells. The NMR relaxation parameters and all the physicochemical quality properties were found to be significantly associated. The findings showed that pre-brining with brine injection followed by brining with low salt levels led to the least denaturation of protein in dry salting and rehydration. Sánchez-Alonso et al. [71] also used LF-NMR spectroscopy for the study of the frozen storage period and improvements in quality of hake frozen in stock for a maximum of 6 months at −10°C. Their results showed that water retention and the apparent values of viscosity decreased, with a higher shear strength representing the usually tougher texture of the hake that formed during storage. This instrument was shown to be efficient for fat, water, and protein analysis, and to provide valuable information on the actions of relaxation and diffusion. NMR spectroscopy was also considered an important tool for the evaluation of fish health, based primarily not only on the fat content but also on chemicals oxidation and the measurement of metabolites [72].

Table 4 summarizes several of the meat, fish, and seafood products that have been identified by using various approaches. Takakura et al. [73] identified the authenticity of flavor in the beef product by using GC-MS. The authenticity of wooden breast disorder in chicken was assessed by Wang et al. [74] and Xing et al. [75] by using H-NMR. Welzenbach et al. [76] investigated the drip loss and association of pork with Single Nucleotide Polymorphisms (SNP) by using GC-MS and LC-MS. Other researchers also have identified some authenticity factors in fish and shrimp by using H-NMR, HPLC, and LC-MS.

### 5.2. Milk and Dairy Products

Precise identification of milk products is only possible if many parameters are studied. Multivariate data analysis (MVDA) can be implemented to discriminate between different variables (e.g., geographical origins, species, etc.) that are related to food authentication. This can be achieved when metabolomics study is combined with chemometric analysis [8]. The breeds or species of milk animals are associated with many milk metabolites. The comprehensive understanding of milk metabolites offers an added advantage in the assessment of milk traits and the detection of milk adulteration. Currently, cheese and yogurt component analyses are also performed through metabolomic approaches, such as GC-MS, NMR, and liquid chromatography-electrospray ionization-MS (LC-ESI/MS).

Polar metabolite profiling by Scano et al. [34] from gas chromatography-mass spectrometry (GC-MS) and MVDA enabled the differentiation of milk typologies. Based on the results, the number of detected cows’ milk errors was around 5%, and the study identified simple-to-detect food fraud and the uniqueness of goats’ milk. Yang et al. [81] also characterized the metabolite profiles of milk obtained from various livestock animals, including cows (Jersey and Holstein), goat, buffalo, horse, camel, and yak. The metabolite differences were identified using non-targeted NMR and LC-MS-based metabolomics approaches. Various metabolites were detected, of which (choline and succinic acid) were used to differentiate Holstein’s milk from that of the other animals observed. The outcome of the study indicated that some of the metabolic pathways were found to be shared by some of the animals. The Jersey cow, goat, buffalo, and yak displayed similar metabolic pathways, including glycerol, glycerophospholipids, leucine, and isoleucine biosynthesis. Meanwhile, non-ruminant animals (horse and camel) have similar unsaturated fatty acid biosynthesis pathways.

Furthermore, this approach is useful for a deeper understanding of how nutritional or quality characteristics contribute to milk composition. Recent developments in milk research metabolomics based on NMR, including applications associating profiling with the nutritional aspects of milk metabolites and applications aimed at connecting milk metabolites with different technical qualities of milk. The new metabolites detected upon profiling are identified and potentially used as biomarkers or as bioactive compounds [82]. In another study, Salzano et al. [33] combined advanced GC-MS identification with metabolite identification in a strong and reproducible technology framework to classify buffalo and mozzarella milk metabolites. In both the milk and the mozzarella cheese, a total of 185 metabolites were consistently found. They used PLS-DA to separate samples of Protected Designation of Origin (PDO) and non-PDO milk and mozzarella. The metabolites were split into two groups by region for the milk samples. The same proportion of certain metabolites (Talopyranose, 2, 3-DI, etc.) was found with the samples of mozzarella. The findings showed that GC-MS and mass spectral databases were a strong medium through which to assess milk and milk-related authenticity. 

Certain parameters can affect milk fermentation and desirable yogurt qualities, such as fermenting conditions, starter crops, and milk characteristics. Trimigno et al. [83] conducted a metabolomic NMR approach to research the yogurt fermentation process and how NMR can detect yogurt quality. The breakdown and a concomitant increase of proteins and lactose during fermentation were also observed. The formate was present at various initial concentrations based on heat processing in the milk, and its time progression differed according to the starting crops: *Lactobacillus* cannot produce the formate but requires it to develop, while *Streptococcus* may convert pyruvate into formate, thus encouraging the symbiotic connection between the two strains. On the other hand, *Lactobacillus* hydrolyzes milk proteins into amino acids, which improves the finished product’s consistency. The NMR based-metabolomics approach revealed that both the species can generate certain metabolites efficiently, including formate or fumarates, and vice versa for amino acids. Table 5 summarizes some milk and dairy products that have been analyzed using different instrumental methods. 

### 5.3. Fruit and Vegetable Product

Consumers have experienced a range of negative events over the last few years, such as unhealthy concentrations of residual pesticides in exported fruit and vegetables, which have contributed to improved knowledge of the geographical origin and food chemistry levels of fruit and vegetables. Organic agriculture, cultivation area, and cultivar are considered the important factors that determine the market value of fruit and vegetables [87]. The primary approach to the detection of fruit and vegetable fraud is to target specific adulteration activities using various analytical platforms by analyzing the macromolecule components of the foods in question, including carbohydrates, amino acids, carotenoids, phenolic compounds, or other organic acids. Different compositions of these phytoconstituents in the fingerprinting analysis may contribute to investigating variations between agricultural systems.

Zhang et al. [88] used the metabolomics approach for berry fruit juice authentication via LC coupled with a quadrupole time-of-flight MS (LC-QTOF-MS). Based on their findings, 41 metabolites, representing three groups of compounds, including anthocyanins, flavonoid, and phenolic acid, were classified in berry juice and its adulterant. Therefore, some of the phenolic compounds found in the targeted metabolic processes might be applied for the authentication and substitution of berry fruit juice. Another study, conducted by Åkerström et al. [89], found the impact of parent plant growth position and origin on anthocyanidin concentrations in bilberries (*Vaccinium myrtillus*). The HPLC analysis showed that the anthocyanidin concentrations varied significantly along with the geographical locations and latitude. The results further showed that the anthocyanidins concentrations in bilberries are well controlled, but also influenced by the climate. They concluded that the diversity of anthocyanidin concentration and composition has a major effect on plant breeders and the potential for future development. Ellagitannin and proanthocyanidin components were examined with LC-MS, MS/MS, and direct infusion-MS, in extracts enriched with tannin made of strawberries, raspberries (*Rubus idaeus*), and cloudberries (*R. chamaemorus*) [90]. The metabolomic method for the evaluation of metabolic changes between different harvesting and maturing conditions of mangosteen (*Garcinia mangostana*) has also been implemented by Anjaritha et al. [91]. The metabolome data obtained from GC-MS were analyzed using multivariate techniques, such as hierarchical clustering, principal component analysis, and partial-to-latent squares analysis. 

A rapid, automatic, high-level throughput and scalable screen to evaluate plant fruit diversity can be established by using all these techniques. NMR technology is also commonly used to understand the variables affecting vegetables, such as tomatoes, leaves, carrots, etc., with their structure, such as origin, variety, irrigation of saltwater, farming techniques, and developmental stages. NMR spectroscopy has been shown to be highly reproducible and non-invasive, and does not require the distinguishing of any compounds from biological mixtures [92].

Table 6 highlights some fruit and vegetable products that have been analyzed by various metabolomics instruments. The authenticity of mangosteen fruit, palm fruit, and lettuce has been analyzed by using GC-MS. Meanwhile, HPLC has been used to detect sugar compounds in palm fruit [93], the nutritional properties of different fruits [94], and the geographical origin of garlic [95].

### 5.4. Other Food Products

Many other food commodities also are being analyzed for their authentication using metabolomics techniques, including honey, coffee/tea, fermented food, etc. Honey adulteration is related to water dilution, sugar, and/or syrup extensions (to the final product or through the feeding of bees with sugar and syrups or artificial honey). Adulterated honey is sometimes branded and marketed as authentic honey, and artificial honey is always mislabeled due to its botanical and geographical origin [8]. Several studies have been conducted to analyze the metabolites in honey using various analytical methods for classification, identification, and authentication purposes. These include chromatographic and MS-based techniques, spectroscopy (FTIR, NMR), stable isotope analysis, flame ionization detectors (FID), sensor arrays, etc. These studies have analyzed and measured the mineral content [98], carbohydrate profile [99], phenolic and flavonoid [100], and aroma compound [101] of honey. 

Furthermore, coffee and tea are also some of the most popular drinks in the world. To demonstrate the quality of these beverages, an efficient and effective metabolomic approach is needed to prevent mislabeling and to monitor fraudulent practices. Jumhawan et al. [102] discovered that upon determining the Asian palm civet coffee (Kopi Luwak) fraction, the metabolomics concept was applied to predict the degree of coffee adulteration using the GC-MS method. Two prediction sets, consisting of approved and commercial coffee, were created with 11 mixes of percentages of a combination of civet and standard coffee. This resulted in a precise estimation of the coffee mixing percentage, successfully validating the composition of the known and unknown samples, and thus quantifying them.

The quality of tea also not only depends on the growing climate but also on its processing technique and geographical origin. Green tea is one of the beverages that are popular due to the health benefits of their nutrients. Some researchers [103,104] have identified green tea by its geographical origin. Different countries of origin in green tea and to a lesser degree in black tea samples were analyzed by PLS-DA data and collected by UHPLC MS. FT-NIR also successfully classified green tea according to its origin with supervised pattern detection. Many researchers used various metabolomic approaches to classify the metabolites that play a major role in the classification of tea grades, as well as other food products.

## 6. Conclusions and Recommendation

This review has shown that many scientific communities have successfully identified a variety of metabolites through food authentication and can benefit greatly from the use of these technologies and approaches. The rapid development of the system and its application for food authentication and adulterant detection may positively impact the global food industry. The use of all these instruments is highly recommended to detect food authentication. As these analyses were detailed and, therefore, expensive, it is suggested that a trial analysis should be performed before carrying out a further analysis of samples. Furthermore, different analytical techniques can be utilized for a single sample to widen the identification scope with a different database as well. Metabolomics provides a wider scope of analytical and detection options for authentication purposes that may improve food quality and preserve food safety. Despite the limitations discussed in some of the technologies involved, various other options are developed and provided to overcome those limitations and to extract the best from the approach. With a wide range of metabolites to be detected, the use of metabolomics with sophisticated technologies, statistical method, and available databases may provide a significant outcome that subsequently leads to a breakthrough in the food authentication process. Continuous revolution in the metabolomics approach will make a major contribution to the food industry in sustaining food safety and quality. Overall, this review provides an overview of metabolomics and the application of its associated technologies in food authentication and the improvement of food authenticity criteria.

## Figures and Tables

**Table 1:** Food authenticity issues for some of the food commodities.

Commodity	Issues	References
Fruits and vegetables	Addition of undeclared ingredients/components (e.g., sugar, water, acid, peel extracts, or pulp wash) into fruit juice. Inaccurate declaration of fruit type.	[10]
Grain	Replacement of non-basmati rice with basmati rice. Durum wheat replacement with common wheat and wheat flour impurities. Distinguishing viable germinating corn and soybean seeds from dead seeds. Inaccurate declaration of cereal rice and wheat geographical and cultivar origin.	[11]
Milk and dairy	Addition of water to milk without any declaration. Mixing of cows’ milk into goat, sheep, or buffalo milk and dairy products (e.g., yogurt, or cheese). Differentiating cheese processed from raw or heat-treated milk. Differentiating milk and cheese based on regions, varieties, and manufacturing processes. Inclusion of melamine or non-milk fat/oil in dairy products. Mislabeling of conventional milk as an organic product.	[12]
Oil and fat	Oil blending without any declaration. Addition of low-quality oils to extra virgin olive oil without any declaration. Adulteration of olive oil with palm oil without any declaration. Adulteration of butter with hydrogenated oil and animal fat.	[10,13]
Meat and fish	Inaccurate declaration of livestock species. Labeling frozen meat as fresh. Addition of water to meat and fish above legally permitted amounts without any declaration. Differentiating fresh and thawed meat.	[10]

**Table 2:** Comparison of benefits and limitations of untargeted and targeted metabolomics [23,24,25,26,27].

Features	Untargeted Metabolomics	Targeted Metabolomics
Benefits	Comprehensive and unbiased. High throughput. Enable discovery of unexpected new compounds in the samples.	Low detection limit. Quantitative analysis. Simpler data interpretation and analysis. Metabolite pathways of the biomarker can be linked once identified.
Limitations	Semi-quantitative. A possible high number of false positives and false negatives. Many detected unknown. Interpretation of data can be difficult.	Limited compounds that can be targeted. Untargeted compounds are not assessed. Quantification requires purified standards of the targeted compounds.

**Table 3:** Benefits and limitations of FTIR spectroscopy.

Instruments	Benefits	Limitations	References
HPLC	Quantitative research is efficient and reliable. Automated operation. Detection with good accuracy. Recovery of quantifiable sample. Convenient for different samples.	No universal detector. Less effectiveness of separation. Harder for beginners.	[37]
FTIR	High sensitivity and high speed. Increase optical throughput. Enable all frequencies that measured metabolites simultaneously. Efficient data interpretation.	Difficulties in analyzing aqueous solution. Cannot identify molecules comprised of two identical atoms symmetric (e.g., N_2_ or O_2_).	[43]

**Table 4:** Metabolomics approach performed on meat, fish, and seafood products.

Food Type	Factors Analyzed	Instrument Used	References
Beef	Flavor	GC-MS	[73]
Chicken	Wooden breast disorder (muscle abnormalities)	H-NMR	[74,75]
Pig	Drip loss (SNP)	GC-MS, LC-MS	[76]
Chicken	Marinade type, storage time, microbial load, sensory	GC-MS	[77]
Beef	Pork adulteration	FTIR	[41]
Fish	Muscle lipid	C-NMR	[78]
Fish	Histamine	HPLC	[79]
Shrimp	Species and geographical origin		[80]

**Table 5:** Metabolomics approach performed on milk and dairy products.

Food Type	Factors Analyzed	Instrument Used	References
Milk	Metabolite profile of different dairy animals	NMR, LC-MS	[81]
	Milk typologies between goat and cow milk	GC-MS	[34]
	Coagulation properties	NMR	[84]
	Metabolic status of the cow	NMR, GC-MS	[85]
	Quality control	NMR	[86]
Yogurt	Fermentation process and quality	NMR	[83]
Cheese	Metabolite profile of buffalo milk and mozzarella cheese	GC-MS	[33]

**Table 6:** Metabolomics approach performed on fruit and vegetable products.

Food Type	Factors Analyzed	Instrument Used	References
Mangosteen fruit	Ripening condition and postharvest treatment	GC-MS	[91]
Palm fruit	Metabolic profile	GC-MS, LC-MS	[32]
Plum fruit	Prediction of individual sugar	HPLC, FT-MIR	[93]
Fruits	Nutritional properties of different fruit types	HPLC	[94]
Garlic	Geographical origin	HPLC-HRMS	[95]
Watermelon	Breeding	NMR	[96]
Lettuce	Metabolite composition	GC-MS, LC-MS	[97]
